# Correction: Unmet needs for analyzing biological big data: A survey of 704 NSF principal investigators

**DOI:** 10.1371/journal.pcbi.1005858

**Published:** 2017-11-13

**Authors:** The PLOS Computational Biology Staff

The URL at the end of the Materials and Methods section directing readers to download the data is incorrect.

The correct URL is: https://figshare.com/articles/Survey_of_biologist_s_computational_needs/4643641

## References

[1] Barone L, Williams J, Micklos D (2017) Unmet needs for analyzing biological big data: A survey of 704 NSF principal investigators. PLoS Comput Biol 13(10): e1005755 https://doi.org/10.1371/journal.pcbi.1005755 29049281 10.1371/journal.pcbi.1005755 PMC5654259

